# Reduced pulmonary function and increased pro-inflammatory cytokines in nanoscale carbon black-exposed workers

**DOI:** 10.1186/s12989-014-0073-1

**Published:** 2014-12-14

**Authors:** Rong Zhang, Yufei Dai, Xiao Zhang, Yong Niu, Tao Meng, Yuanyuan Li, Huawei Duan, Ping Bin, Meng Ye, Xiaowei Jia, Meili Shen, Shanfa Yu, Xiaofa Yang, Weimin Gao, Yuxin Zheng

**Affiliations:** National Institute for Occupational Health and Poison Control, Chinese Center for Disease Control and Prevention, 29 Nanwei Road, Beijing, 100050 China; Department of Toxicology, School of Public Health, Hebei Medical University, Shijiazhuang, China; Henan Provincial Institute for Occupational Health, Zhengzhou, China; Jiao Zuo Center for Disease Control and Prevention, Jiaozuo, China; Department of Environmental Toxicology, The Institute of Environmental and Human Health, Texas Tech University, Lubbock, TX 79409 USA

**Keywords:** Carbon black, Nanoparticles, Occupational exposure, Pro-inflammatory cytokines, Pulmonary function

## Abstract

**Background:**

Although major concerns exist regarding the potential consequences of human exposures to nanoscale carbon black (CB) particles, limited human toxicological data is currently available. The purpose of this study was to evaluate if nanoscale CB particles could be responsible, at least partially, for the altered lung function and inflammation observed in CB workers exposed to nanoscale CB particles.

**Methods:**

Scanning Electron Microscopy (SEM), Transmission Electron Microscopy (TEM), and Brunauer-Emmett-Teller were used to characterize CB. Eighty-one CB-exposed male workers and 104 non-exposed male workers were recruited. The pulmonary function test was performed and pro-inflammatory cytokines were evaluated. To further assess the deposition and pulmonary damage induced by CB nanoparticles, male BALB/c mice were exposed to CB for 6 hours per day for 7 or 14 days. The deposition of CB and the pathological changes of the lung tissue in mice were evaluated by paraffin sections and TEM. The cytokines levels in serum and lung tissue of mice were evaluated by ELISA and immunohistochemical staining (IHC).

**Results:**

SEM and TEM images showed that the CB particles were 30 to 50 nm in size. In the CB workplace, the concentration of CB was 14.90 mg/m^3^. Among these CB particles, 50.77% were less than 0.523 micrometer, and 99.55% were less than 2.5 micrometer in aerodynamic diameter. The reduction of lung function parameters including FEV1%, FEV/FVC, MMF%, and PEF% in CB workers was observed, and the IL-1β, IL-6, IL-8, MIP-1beta, and TNF- alpha had 2.86-, 6.85-, 1.49-, 3.35-, and 4.87-folds increase in serum of CB workers, respectively. In mice exposed to the aerosol CB, particles were deposited in the lung. The alveolar wall thickened and a large amount of inflammatory cells were observed in lung tissues after CB exposure. IL-6 and IL-8 levels were increased in both serum and lung homogenate.

**Conclusions:**

The data strongly suggests that nanoscale CB particles could be responsible for the lung function reduction and pro-inflammatory cytokines secretion in CB workers. These results, therefore, provide the first evidence of a link between human exposure to CB and long-term pulmonary effects.

**Electronic supplementary material:**

The online version of this article (doi:10.1186/s12989-014-0073-1) contains supplementary material, which is available to authorized users.

## Background

Inhalation of particulate matter (PM) from fuel combustion is associated with adverse health effects, including reduced lung function [1] and increased mortality [2]. Fuel-derived PM in the inhalable size range (<10 μm in aerodynamic diameter, PM10) is dominated by aggregates of nanoparticles of carbon black (CB) [3]. CB has been suggested to serve as a valuable additional air quality indicator and would be particularly useful to evaluate health risks of air pollution dominated by primary combustion emissions and the benefits of traffic abatement measures [4]. CB is the powder that is nearly pure carbon manufactured by the controlled vapor-phase pyrolysis of hydrocarbons and consists of primary particles with diameters smaller than 100 nm in all three dimensions. This form of CB is an amorphous carbon that has a high surface area to volume ratio [5]. CB is widely used because of its characters of sluggishness and stability. It is the conventional filler in rubber to achieve the grip and mechanical stability of tires. CB ranks as one of the top 50 industrial chemicals worldwide and has been manufactured for decades in quantities in the range of megatons per year [6].

Because of its unique characteristics, CB has served as a negative control to explain some toxicity of other nanoparticles [7]. However, evidence indicated that CB nanoparticles could induce lung inflammation and histopathological injury [8-10]. International Agency for Research on Cancer (IARC) classifies CB as a possible carcinogen to humans [11]. The US epidemiological studies showed no excess of lung cancer in CB workers [12-14]. A UK study showed a marginally significant increase of lung cancer in CB production workers [15], however, the follow-up investigation demonstrated that this increase was not related to cumulative CB exposure, but a combination of factors including prevalence of smoking, previous occupations, social class, and regional effects unrelated to smoking habits [16]. They reported a 73% increase in lung cancer mortality among CB production workers when compared with the general population (Standardized mortality ratios [SMR] 173, 95% CI 132 to 222). In German CB workers, the mortality from lung cancer was more than 2-fold increase in the main (census) cohort (SMR 218, 95% CI 161 to 287) and nearly a threefold increase (SMR 289, 95% CI 206 to 394) among the workers hired after 1960. The high lung cancer SMR could not be fully explained by selection, smoking, or other occupational risk factors, but these results also provided little evidence for an effect of CB exposure [17]. Therefore, the definitive evaluation of CB-induced carcinogenic effects is still insufficient and more evidence from epidemiological studies in human is required to understand the adverse effects of CB.

It is known that a greater and more persistent inflammatory and related oxidative stress response is a likely mechanism by which high lung burdens of particles can induce genotoxicity resulting in epithelial cell mutations and subsequent lung tumor formation [18,19]. However, the lung inflammatory and lung function deficiency caused by burdens from CB exposure is still controversial. There is very limited evidence of lung inflammatory response induced by CB in human beings. In fact, in the literatures about the CB-induced lung diseases, none of them analyzed workers solely exposed to CB [9]. In the present study, the CB packers were exposed to acetylene black, in which no other particles and organic chemicals were formed. These CB packers were therefore investigated to explore the effects of CB on lung functions and inflammation.

Industrial CB is produced within a closed reactor where the primary particles form aggregates and then become the indivisible entities of CB. Following nanoparticles inhalation, the deposition characteristics in the lungs, the potential toxic effects induced, and the toxicokinetic fate is predominantly determined by the agglomeration status of nanoscaled primary particles. Based on the results of various approaches, a tendency of nanoscaled particles to form larger size agglomerates following deposition and interaction with cells or the respiratory tract is predominant. In order to explain the relationship between the characteristics of CB (size and dose) and lung damage and inflammation in CB workers, the physical-chemical characteristics and the concentration of CB were detected in the CB-exposed occupational environment in the present study. Because it is difficult to further study the deposition characteristics in the lungs following CB inhalation and the follow-up changes of lung inflammation in the CB-exposed workers, the animal inhalation experiment could be pursued to mimic the exposure condition of CB workers. The hypothesis of this study is that lung functional impairments and pro-inflammatory cytokines release are associated with occupational inhalation exposure to CB. Furthermore, the deposition of CB particles will induce histopathological alteration of the lungs as well as the pro-inflammatory cytokines release in mice after CB inhalation.

## Results

### Characterization of CB

**Figure 1:**
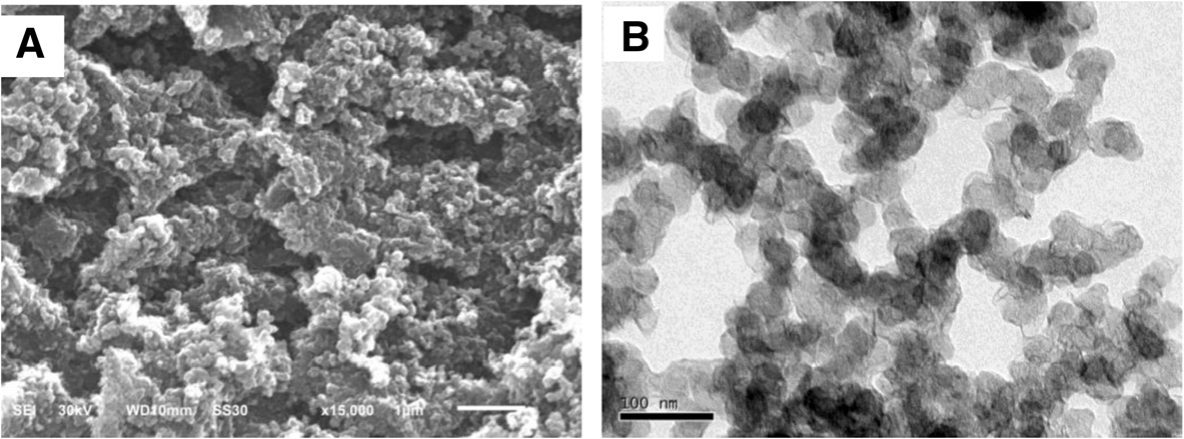
**Scanning electron microscopy (A) and transmission electron microscopy (B) images of CB.** The CB powder was deposited on 200-mesh copper grids. The particle primary structure was 30–50 nm and globular.

The expected three-dimensional nanostructure of CB is clearly confirmed by the Scanning Electron Microscopy (SEM) and Transmission Electron Microscopy (TEM). CB has a high carbon purity (>99.8%) and consists of globular shaped particles. Agglomerates of tens to hundreds of nanometer were formed, but no intermediates structure was observed (Figure 1 and Table 1). SEM examinations (Figure 1A) indicated that particles ranged from 30 to 50 nm in size. The larger particles exhibited numerous spherical protuberances on the surfaces, suggesting that they were formed during the spray drying process through fusion of the much smaller particles. TEM confirmed that the CB particles contained clusters comprised of smaller particles, 30 to 50 nm in size (Figure 1B). The iodine absorption value of CB will not fall below 90 (g/Kg). Brunauer-Emmett-Teller (BET) measurements of the CB particles resulted in a surface area of 74.85 m^2^/g. The diameter analysis showed that about 99.55% particles are <2.5 μm and more than 50% particles are <0.523 μm in this workplace (Table 2). The small size, along with the high surface area of the particles, tends to induce strong biological activities.

**Table 1:** **Physicochemical parameters of the CB**

**Physicochemical parameters**	**Carbon black**
Particle size distribution (SEM and TEM)	30-50 nm, globular
State of agglomeration	200-400 nm
Dispersibility	agglomerated
In water	200-400 nm
In tween-80 (0.04%)	30-50 nm
BET	74.85 m^2^/g
Zeta Potential	−15.37 (MV)

**Table 2:** **The distribution with different size of CB particles in the workplace (particles/cm** ^**3**^ **)**

**Size**	**Mean**	**Maximum**	**Minimum**	**SD**	**Percentage (%)**	**Accumulative percentage (%)**
<0.523 μm	233.77	270.6	208.2	24.32	50.77	50.77
0.523-1 μm	211.33	220.86	206.52	5.49	45.90	96.67
1-2.5 μm	13.24	13.71	12.27	0.58	2.88	99.55
2.5-20 μm	2.08	2.24	1.95	0.13	0.45	100.00

### Characterization of the workers and CB exposure

The average age, body mass index (BMI), smoking and drinking habit, duration of exposure, and work shift status before the physical test were not significantly different between the control and CB exposure groups (Table 3). Stationary samplers and personal air samplers carried by volunteers were used to detect the CB concentrations in the air of the CB workplace. The mean atmospheric concentration of CB was 1.63 mg/m^3^ by the stationary samplers in worksites. However, the mean concentration of CB was 14.90 mg/m^3^ measured by personal air samplers from 15 volunteers, which is nearly 4.26-fold higher than the current Threshold Limit Value (TLV) of 3.5 mg/m^3^ [20]. Although local exhaust ventilation systems are installed, the workers were likely to be exposed to high concentrations of CB dusts due to their reluctance to wear the masks.

**Table 3:** **The characteristics of workers in the control and CB-exposed groups**

**Variables**	**Control group (n = 104)**	**CB-exposed group (n = 81)**	***P***
Age (years, mean ± SD)	44.5 ± 5.94	45.9 ± 5.42	0.108^a^
BMI (kg/m^2^, mean ± SD)	25.0 ± 3.30	25.1 ± 3.93	0.887^a^
Current smoker, yes/no (% yes)	68/36 (65.4)	56/25 (69.1)	0.590^b^
Pack years of smoking	17.87 ± 12.21	13.20 ± 8.92	0.019^a^
Never smoking (% never)	31/73(29.8)	22/59(27.2)	0.693^a^
Alcohol user, yes/no (% yes)^c^	15/87 (14.7)	12/69 (14.8)	0.984^b^
Working year (years, mean ± SD)	---	12.5 ± 11.07	---
Work shift before the physical test, at work/at rest (% at work)	84/20 (80.8)	72/9 (88.9)	0.150^b^
Night shift within last week, yes/no (% yes)	80/24 (76.9)	65/16 (80.0)	0.558^b^

^a^ *t*-test was used to compare values from both groups. Differences were considered significant when *P <* 0.05. ^b^Chi square test was used to compare values from both groups. ^c^Two missing values in the control group. Differences were considered significant when *P <* 0.05.

### The clinical symptoms and lung functions of the workers

All the subjects in the CB exposure group have cough or chronic sputum production but there is no changes in the image of chest x-rays. The results of lung function tests were shown in Table 4. Exposure to CB was associated with a significant reduction in percent predicted forced expiratory volume in 1 second (FEV1%), FEV1/forced vital capacity (FVC), percent predicted maximal midexpiratory flow curve (MMF%) and percent predicted peak expiratory flow (PEF%) when compared with the control group. The means of FEV1%, PEF%, and MMF% had 5.54, 15.26, and 9.79 percent decrease in the CB exposure group compared with the controls, respectively. Whereas, the level of percent predicted forced vital capacity (FVC%) was not significantly different between the CB exposure group and the control group. Nevertheless, no subject was clinically diagnosed with chronic obstructive pulmonary disease (COPD) or asthma in either of the CB exposure or control groups. Smoking is a major confounder for the determined pulmonary parameters, therefore the stratification was used to test for differences in pulmonary parameters. When stratified by smoking status, FEV1% and MMF% were not significantly different between the CB exposure and control groups. The FEV1/FVC was significantly lower in CB-exposed workers with pack-years ≤ 12.5 than that of the control (*P <* 0.05). Multiple linear regression analysis showed that there was a statistically significant association between exposure to CB and FEV1/FVC, MMF%, and PEF% after adjusting for the important confounders, including age, weight, height, smoking, and drinking habit (Additional file 1: Table S1). There was no interaction between smoking and FEV1%, FVC%, FEV1/FVC, MMF%, and PEF% values (Additional file 2: Table S2).

**Table 4:** **The pulmonary function indexes in control and CB-exposed groups (mean ± SD)**

**Variables**	**Control group (n = 104)**	**CB-exposed group (n = 81)**	***P*** **-value**
FEV1 (%)	103.61 ± 14.52	98.07 ± 13.53	0.019^a^
FVC (%)	104.67 ± 14.64	100.12 ± 13.47	0.071^a^
FEV1/FVC	0.87 ± 0.05	0.84 ± 0.05	0.001^a^
PEF (%)	93.76 ± 17.86	78.50 ± 16.80	<0.001^a^
MMF (%)	96.45 ± 23.18	86.86 ± 23.06	0.001^a^

^a^Two-sample *t* test. Differences were considered significant when *P <* 0.05. FVC%: percent predicted forced vital capacity, FEV1%: percent predicted forced expiratory volume in 1 second, MMF%: percent predicted maximal midexpiratory flow curve, and PEF%: percent predicted peak expiratory flow.

### The pro-inflammatory cytokines levels in serum of the CB workers

Table 5 showed the pro-inflammatory cytokines levels in serum of the workers detected by CBA. The levels of the interleukin 1β (IL-1β), interleukin 6 (IL-6), interleukin 8 (IL-8), macrophage inflammatory protein-1β (MIP-1β), and tumor necrosis factor α (TNF-α) significantly increased in the CB exposure group compared with the control group (*P <* 0.05). The medians of IL-1β, IL-6, IL-8, MIP-1β, and TNF-α had 2.86-, 6.85-, 1.49-, 3.35-, and 4.87-folds increase in the CB exposure group compared with the control workers. The level of monocyte chemoattractant protein-1 (MCP-1) was not significantly different between the CB exposure group and the control group. When stratified by smoking status, there was no interaction between smoking and IL-1β, IL-6, IL-8, MIP-1β, and TNF-α levels (Additional file 3: Table S3). The multiple regression analysis of age, BMI, smoking, alcohol drinking, and CB exposure on serum cytokines is shown in Table 6. There was a statistically significant association between exposure to CB and the levels of IL-1β, IL-6, IL-8, MIP-1β, and TNF-α in the serum of workers (*P <* 0.01). Statistically significant associations were also observed between BMI and the level of IL-8 (*P <* 0.05) and between age and the levels of MIP-1β and TNF-α (*P <* 0.01). No significant associations were observed between smoking and drinking habits and the levels of all the above cytokines.

**Table 5:** **The cytokines levels of control and CB workers**

**Cytokines**	**Control group (n = 104) pg/mL, median (5%-95%)**	**CB-exposed group (n = 81) pg/mL, median (5%-95%)**	***P*** **value**
IL-1β	4.16 (0.00-17.75)	11.88 (1.98-38.08)	<0.001^a^
IL-6	27.51 (2.16-180.18)	188.32 (46.13-643.16)	<0.001^a^
IL-8	746.30 (163.55-1879.01)	1117.10 (369.36-3737.82)	<0.001^a^
MIP-1β	804.09 (225.35-2888.59)	2694.52 (1136.97-10074.81)	<0.001^a^
TNF-α	47.75 (0.00-191.33)	232.36 (76.47-572.05)	<0.001^a^
MCP-1	254.75 (94.29-428.72)	238.76 (92.39-438.51)	0.242^a^

^a^ *t*-test was used to compare values from both groups. Differences were considered significant when *P <* 0.05.

**Table 6:** **Multiple regression analysis (regression coefficient and 95% CI) of age, BMI, smoking, alcohol drinking, CB exposure and serum cytokines** ^**a**^

**Parameters**	**IL-1β**	**IL-6**	**IL-8**	**MIP-1β**	**TNF-α**	**MCP-1**
Age	−0.015 (−0.039 - 0.008)	−0.027 (−0.055 - 0.001)	0.001 (−0.017 - 0.018)	−0.036 (−0.055 - -0.018)**	−0.041 (−0.071 - -0.012)**	0.017 (0.001 - 0.033)*
BMI	−0.008 (−0.044 - 0.028)	0.012 (−0.033 - 0.056)	0.032 (0.004 - 0.060)*	0.015 (−0.014 - 0.044)	0.016 (−0.030 - 0.062)	−0.031 (−0.058 - -0.003)*
Smoking	−0.050 (−0.336 - 0.236)	−0.003 (−0.342 - 0.336)	0.161 (−0.050 - 0.373)	−0.075 (−0.297 - 0.147)	0.093 (−0.263 - 0.449)	0.042 (−0.150 - 0.234)
Drinking	−0.048 (−0.432 - 0.336)	−0.030 (−0.483 - 0.423)	0.102 (−0.181 - 0.384)	−0.062 (−0.366 - 0.243)	−0.109 (−0.588 - 0.369)	0.100 (−0.166 - 0.366)
CB exposure	0.680 (0.412 - 0.949)**	1.924 (1.604 - 2.245)**	0.659 (0.459 - 0.860)**	1.301 (1.091 - 1.512)**	1.772 (1.436 - 2.109)**	−0.015 (−0.095 - 0.065)

^a^Ln-transformed. *P < 0.05, and **P < 0.01.

### The clinical observation of the animals and the changes in body weight and organ coefficient

Mice were exposed to CB particles for 7 and 14 d by inhalation. There was a slight reduction in activities and diet of mice after inhalation of CB for 7 and 14 d. The activities and food intake of mice returned to normal after 14 d. There were no other changes in clinical observation of animals. In both the CB treatment and control groups, the weights of mice were slightly decreased after a 7 d exposure whereas all the mice gained weight slowly after a 14 d exposure and a recovery for another 14 d. No significant difference of weight was observed between the control and treatment groups (Additional file 4: Figure S1). The lung and spleen coefficient in the 14 d exposure group were significantly increased compared with the control (P < 0.01). The coefficient of the other organs (trachea, liver, and kidney) did not differ significantly between the treated and control groups (Additional file 5: Table S4).

### The cytokines levels in serum and lung homogenate in mice

**Figure 2:**
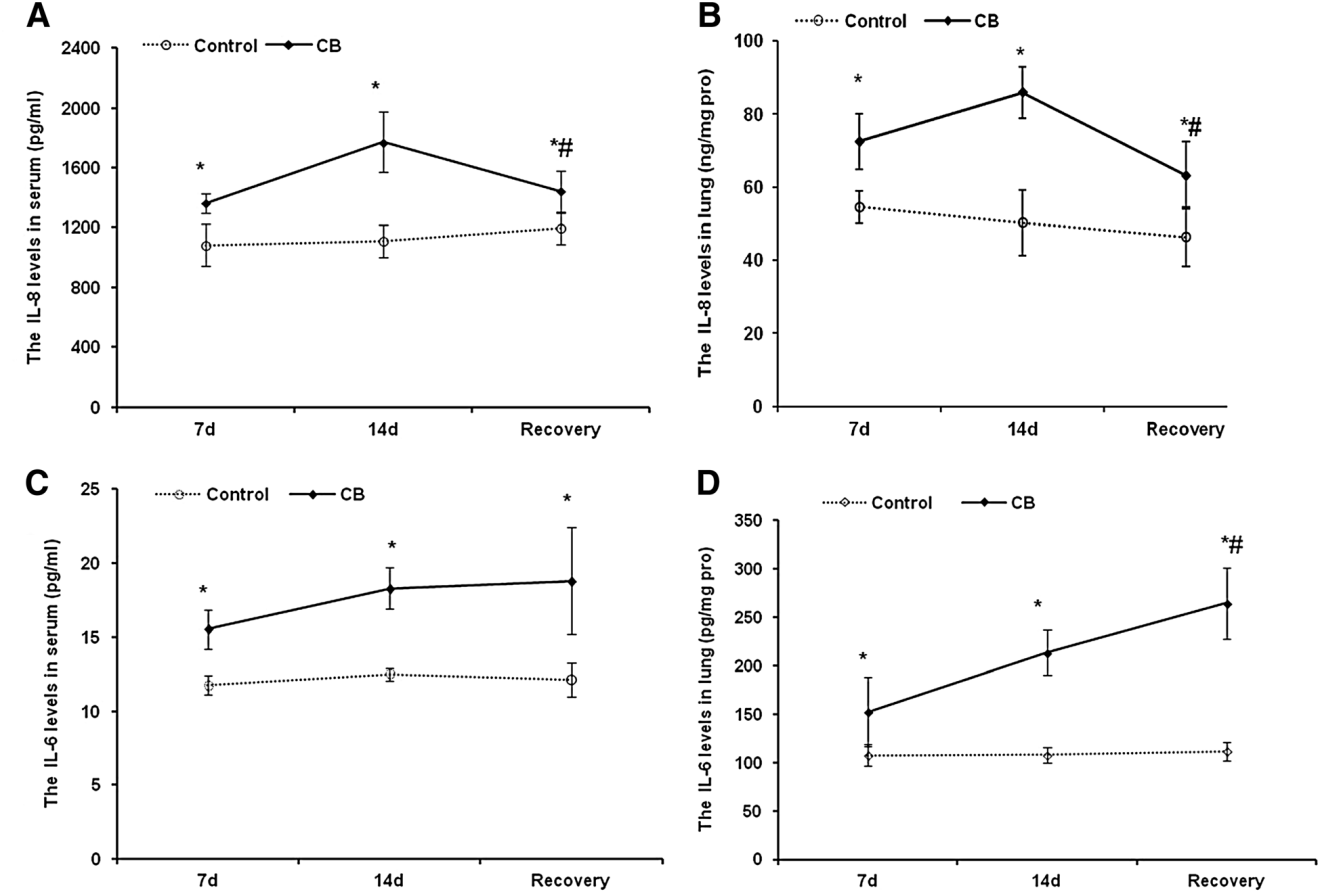
**The levels of IL-8 and IL-6 in serum and the lung tissue of mice after CB exposure for different time and recovery for 14 d after 14 d CB exposure. A**: The levels of IL-8 in serum of mice. **B**: The levels of IL-8 in the lung tissue of mice. **C**: The levels of IL-6 in serum of mice. **D**: The levels of IL-6 in the lung tissue of mice. Data were expressed as mean ± SD. Multi-group comparisons of the means were carried out by a one-way analysis of variance test followed by SNK’s multiple comparison tests. * *P <* 0.05 compared to the control group.

**Figure 3:**
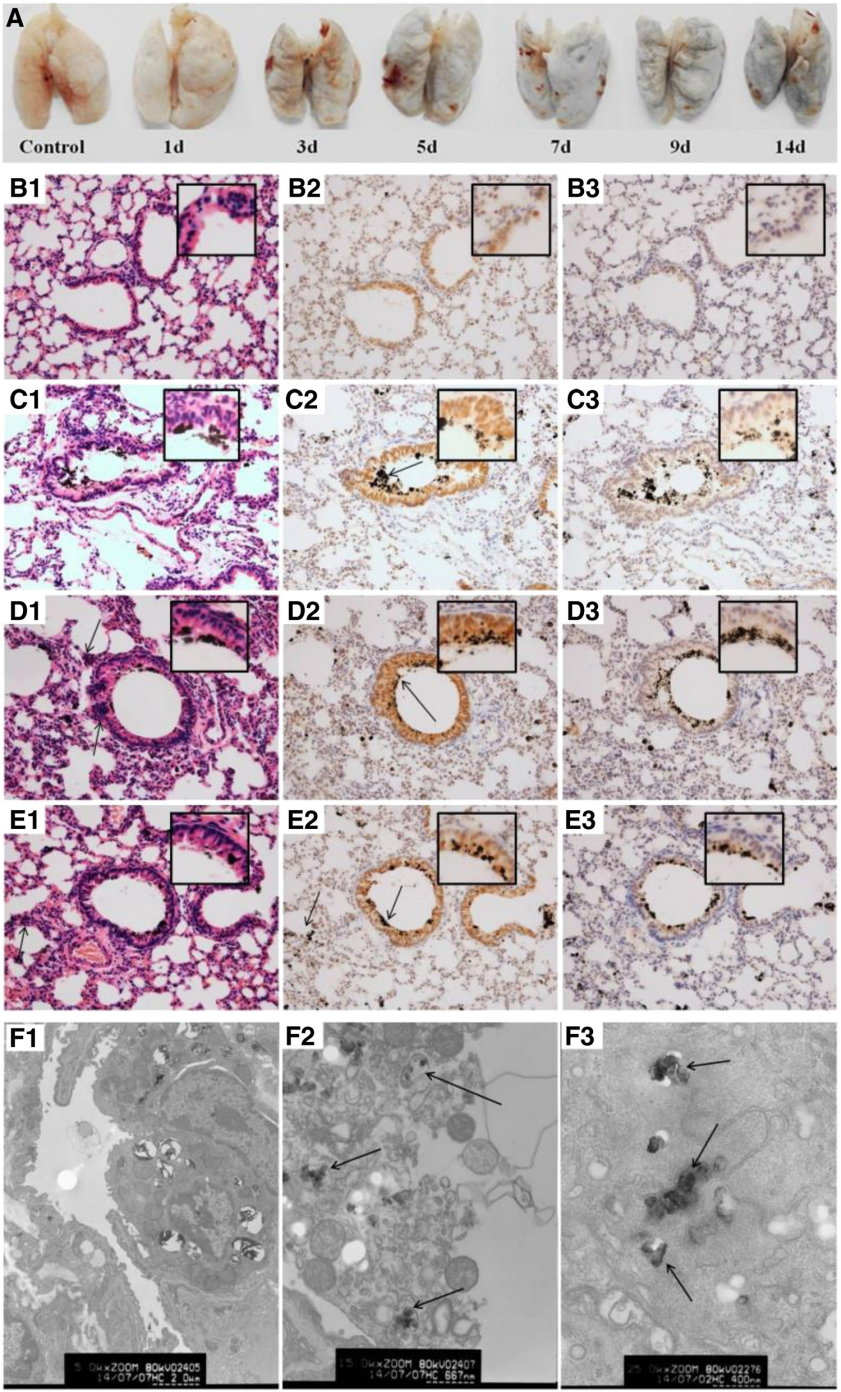
**Images of the lung tissue in mice and the IL-8 and IL-6 expression in the lung tissue of mice by immunohistochemical staining. A**: Images of the lung tissue in mice after CB inhalation for 14 d. The mice were deeply anesthetized with chloral hydrate and perfused by injection from the left ventricle with 20 mL of 37°C saline solution and then the lung tissues were separated. **B1-E1**: Histopathology of the lung tissue in mice after exposure to CB particles for different time by HE staining (200×). The arrows in **C2**, **D2**, and **E2** indicate the CB particles in pulmonary alveoli or bronchioli. The arrows in **D1** and **E1** indicated inflammatory cells. **B2-E2**: The IL-8 expression in lung cells after CB exposure for different time points by immunohistochemical staining (200×). The IL-8 positive cells displayed brownish yellow granules. In lung cells, IL-8 was located mainly in the cytoplasm and karyon. **B3-E3**: The IL-6 expression in lung tissue after CB exposure for different time points by immunohistochemical staining (200×). In lung cells, IL-6 was a granular brown substance located mainly in the cytoplasm and karyon. **B1-B3**: Control group; **C1-C3**: 7 d CB exposure group; **D1-D3**: 14 d CB exposure group; and **E1-E3**: recovery for 14 d after 14 d CB exposure. Inset: a higher magnification of the lung tissue (400×). **F**: TEM images of lung cells in mice after CB inhalation for 14 d. **F1**: Alveolar type II epithelial cells in control (5000×); **F2**: The disintegration of the macrophages in the lung of mice after CB exposure for 14 d (15000×) (Staining by uranyl acetate and lead citrate); and **F3**: The CB particles in a lung macrophage (25000×) (No uranyl acetate and lead citrate staining). The arrows indicate the CB particles in a lung macrophage.

The changes of pro-inflammatory cytokines levels in serum and lung homogenate or lung tissue of mice were detected by ELISA or immunohistochemical staining (IHC), respectively. As shown in Figure 2, the levels of IL-6 and IL-8 significantly increased in the 7 and 14 d CB exposure groups compared with the control groups (*P <* 0.05). However, no significant changes were observed for the levels of the IL-1β, MIP-1β, and TNF-α in CB exposure groups compared with the control groups (data not show). In the recovery group, the level of IL-6 in lung homogenate increased compared with the 14 d CB exposure group and the control group (*P <* 0.05). In addition, the level of IL-8 in serum and lung homogenate increased compared with the control group (*P <* 0.05), but decreased compared with the 14 d CB exposure group (*P <* 0.05). From the images of IHC of lung tissues shown in Figure 3, the positive staining cells for IL-8 and IL-6 in the control group tended to be localized to the basal lamina of epithelial cells and in close proximity to the musculature of the vessels or airways, while there was little staining in the alveolar epithelial cells. In mice treated with CB for 7 and 14 d, there was a clear increase in the positive staining for IL-8 and IL-6 around airways and bronchium (Figure 3C2, C3, D2, and D3). In the recovery group, the IL-8 and IL-6 positive cells were similar to the CB exposure groups, but with less positive alveolar epithelial cells.

### Histopathological changes of the lung tissue in mice

The appearance of lung tissue in the CB exposure group became black while the lung in the control group was white (Figure 3). The histological photomicrographs of the lung were shown in Figure 3B1-E1. In lung tissue, CB particles were deposited in the pulmonary alveoli or bronchioli after CB exposure for 7 and 14 d. In the recovery group, there were still some CB particles deposited in the bronchioli but more particles in the pulmonary alveoli and the alveolar wall tissue, whereas the weight of the lung decreased. After CB exposure for 7 and 14 d, the alveolar wall thickened significantly and a large amount of inflammatory cells, constituted mainly by lymphocytes, were infiltrated. The characteristic features of the pulmonary alveoli and the bronchioli were slightly disarranged and swollen with some of the cells showing vacuolar degeneration in the CB exposure groups (Figure 3C1 and D1). The score of the lung abnormalities was shown in Additional file 6: Figure S2. The scores in exposure groups (7 and 14 d) were significantly higher than that of the control (P < 0.01). This histological change remained in the recovery group (Figure 3E1) and the score in the recovery group was still significantly higher than that of the control (P < 0.01), but there were no significantly different between CB exposure for 14 d group and the recovery group.

Ultrastructure of the lung was detected by TEM (Figure 3F). The image of TEM demonstrated particles in the alveolar space and macrophages. For removing the effects of uranyl acetate and lead citrate staining, we observed no staining of the particles in macrophages (Figure 3F). In alveolar macrophages, there was an increase of primary and secondary lysosome and broken mitochondrial cristae. In the secondary lysosome, there were some insoluble particles that had the same electron density as the CB particles shown in Figure 1B. The pulmonary interstitial hyperplasia and collagen fiber hyperplasia in alveolar septa were observed. In alveolar type II epithelial cells, the microvillus and the osmiophilic multilamellar body disappeared, perinuclear spaces widened, and organelles reduced. These ultrastructure damages indicated the functional deficiency of the alveolar type II epithelial cells.

## Discussion

CB has been studied extensively as to its toxicology, especially as an example of a less toxic, low soluble particle with no considerable harm compared to organics or metals. In long-term animal studies, CB was found to be a carcinogen in rat and the burden of CB in the lung was very likely to play an important role in this affect [21]. In studies of the health status of individuals working in the CB industry, there is evidence of abnormalities in chest radiographs and respiratory morbidity, but equivocal findings were reported about its relationship to lung cancer [16,22,23]. However, none of these studies analyzed a worker population exposed solely to CB, and there are no existing data to explain the health effects in human beings [9]. In the present study, the subjects were exposed to CB in acetylene black workplace. In the process of production of acetylene black, there are no other particles and chemicals, such as polycyclic aromatic hydrocarbons (PAHs), existing in the occupational environment where the packers work. Therefore, the subjects who participated in the present study were solely exposed to CB. They were surveyed and examined for physical and biochemical parameters. Moreover, given the tissue localization and the effects of CB particles on mice lung tissue, the data here strongly suggests that CB particles could be responsible, at least in part, for the pulmonary function reduction and inflammatory response observed in CB workers.

The sizes of particles can affect their abilities to enter the lower respiratory tract and induce impaired pulmonary function. CB particles in nano-size also exhibited a higher propensity of inducing cytotoxicity, inflammation, and altered phagocytosis in human monocytes than their micron size [24]. Generally, the basic building blocks of CB include primary particles, aggregates, and agglomerates. The aggregates can result in agglomerates form, which are the typical form of CB in commerce. The aggregates and agglomerates of CB are extremely fluffy and fine powders and have a large surface area with aggregate dimensions ranging from ten to a few hundred nanometers. The main concern about exposure to CB is the ultrafine size of the primary particle (10–500 nm) and their aggregates (80–800 nm). Very limited research has been done to evaluate the size distribution of the agglomerates in occupational environments, and we analyzed the size distribution of CB in the workplace (Table 1 and Figure 1). The results of SEM and TEM showed that the CB particle in this study was 30–50 nm with a globular shape. Some of them were aggregates. Only 0.45% of particles are >2.5 μm in the workplace and more than 50% of the CB particles are <523 nm (Table 2). The small size of particles, and often with a high surface area, might cause inflammation and lung epithelial hyperplasia [24,25]. In our study, the surface area of the CB particle is 74.85 m^2^/g. In contrast to the high surface area (213 m^2^/g) CB, low surface area CB (37 m^2^/g) has been shown to induce less severe inflammatory changes on a mass-based dose [8]. In a carcinogenicity study, exposure of female rats to 6 and 12.2 mg/m^3^ CB (particles size 15 nm, surface area 213 m^2^/g) for two years led to an increased tumor incidence in the lung [26]. However, in another carcinogenicity study, CB with a low surface area (43 m^2^/g) caused concentration-related increase of lung weight and polymorpho-nuclear neutrophils, biochemical changes of broncho-alveolar lavage fluid (BALF), and increased incidence of adenomas and adenocarcinomas in rats [27]. In the present study, inhalation of 30 mg/m^3^ CB particles for 14 d caused a temporary increase in lung weight (Additional file 2: Table S2). These results indicate that the surface area might not be a single crucial factor of CB toxicity, at least for carcinogenesis.

There are many concerns about the adverse effects of chronic exposure to CB, but the possible effects of CB exposure on pulmonary function have not been thoroughly investigated and are subject to debate and controversy. Acute intense CB exposure can cause respiratory symptoms and an obstructive ventilator defect [28]. Perennial CB exposure was associated with a significantly increased prevalence of respiratory symptoms and acute, partially reversible, and chronic irreversible significant decrease in some parameters of pulmonary function, with a spirometric pattern consistent with restrictive ventilatory disorders [29]. However, in reports from CB plants in the US [30] or Germany [31], there was no significant associations observed between CB exposure and reduced lung function. Some other studies revealed that the pulmonary function parameters of CB-exposed workers were significantly decreased, but the decrease was related to the effects of smoking and age using multiple regression analysis [30,32]. In the present study, all of the exposure workers have respiratory symptoms including cough and sputum production because they exposed to the high concentration of CB particles. Though FEV1/FVC and PEF(%) parameters of pulmonary function were significantly decreased in CB workers compared with the control workers, none of the exposure and non-exposure workers have pathologically low values of parameters of pulmonary function in this study. In mild asthma patients 40–59 years old, the FEV1% values were >80% [33], while we found the mean of FEV1% was 98.07%, which is far from being diagnosed with asthma. Generally, for the asthma patients, <40% predicted FEV1 or PEF indicates severe asthma exacerbation and ≥ 70% predicted FEV1 or PEF is a goal for discharge from the asthma emergency care setting. In this study, the PEF% in CB exposure workers was 78.5%, which is more than the value of the criteria of discharge from the asthma emergency care setting. After adjusting for important confounders, including age, weight, height, smoking, and drinking habit, significant associations were still present between reduction in most parameters of pulmonary function and CB exposure. Our results are similar to other studies that had been conducted in tire manufacturing and rubber factories [29,34,35]. In the present study, the mean concentration of CB was 14.90 mg/m^3^ by 15 volunteers carrying personal air samplers, which is nearly 4.26-fold higher than the current TLV of 3.5 mg/m^3^. The actual amount of CB exposure of the packers is about 10-fold higher than that in the atmosphere of packing workshop. Neghab *et al.* [29] reported that CB exposure at concentration of 2-fold exceeding TLV was associated with a significant increase in respiratory symptoms, including cough, sputum production, wheezing, and dyspnea and with decreases in both FVC and FEV1 in CB-exposed workers. Harber *et al.* [36] found that CB exposure at a low concentration (0.5 mg/m^3^), which were 7-fold lower than the TLV, only caused small reductions in the FEV1 but not in the other spirometic parameters. In this study, the FEV1%, FEV1/FVC, PEF%, and MMF% were significantly reduced in CB-exposed workers. In view of these differences between the literature and our results, the CB exposure dose might be an important factor. However, Wellmann *et al.* reported that the estimated cumulative CB exposure was negatively associated with lung cancer mortality and there was no significantly increased risk with one of the simple measures of employment in work areas with high CB exposure. These results could be explained with healthy worker effects and the unclear latency period for exposure to CB and the development of lung cancer [17]. Though our results may support the hypothesis that CB exposure at a high concentration induces the lung function deficiency in CB-exposed workers, further analyses looking at time dependent factors and CB occupational exposures in more detail are required.

Although the mechanism for PM-induced health effects is not fully defined, the persistent inflammatory and related oxidative stress might play important roles. However, there was no direct evidence for the inflammatory mechanism induced by sole CB in human beings. In the present study, the increases of pro-inflammatory cytokines, including IL-1β, IL-6, IL-8, MIP-1β, and TNF-α, were observed in the serum of CB-exposed workers. Multiple logistic regression analyses showed an association between CB exposure and the increase of cytokines after adjusting for important confounders, including age, BMI, smoking, and drinking habit. Our study is the first to provide persuasive evidence for CB-induced pro-inflammatory cytokines in human beings. Therefore, these pro-inflammatory cytokines might be useful biomarkers of pulmonary inflammatory in the workers who exposed to CB at a high concentration.

Some animal experiments and *in vitro* studies supported that CB exposure could induce the inflammatory response. For instance, after rats were exposed to 116.4 nm CB for 4 weeks at a nominal concentration of 15.6 ± 3.5 mg/m^3^, IL-6 level in serum and IL-6 mRNA in lung were markedly elevated. Although a severe form of alveolar inflammation or fibrosis was not observed in rats exposed to CB, it was certain that CB exposure induced a mild inflammatory response in the lung [37]. Vesterdal *et al.* [38] treated mice with CB nanoparticles using intratracheal instillation and found that MCP-1 was increased 24 h after instillation. Stoeger *et al.* [39] also reported a dose-dependent inflammatory response by measuring the number of neutrophils, IL-1β, and MIP-1β in lavage fluid of mice after exposure to ultrafine CB for 24 h. In addition, CB was able to cause detectable but low level pro-inflammatory effects in rats following 7 h inhalation exposure [40]. In human bronchial epithelial cell line (16HBE14o-), 13 nm CB particles have been shown to cause increases of IL-6 and TNF-α [41]. However, there were still some opposite conclusions in animal inhalation experiments. For example, inhalation of CB particles with a diameter of 83.3-87.9 nm for 4 weeks neither induced toxicity of the rat’s lungs nor elicited most of pulmonary pro-inflammatory cytokines. The histological analysis further revealed that nanoscale CB particles did not induce inflammatory responses in the respiratory system [42]. Another study indicated that a short-time inhalation of nanoscale CB particles at 10 mg/m^3^ could not induce changes of IL-6, IL-8, and TNF-α in rat [43]. In the present study, the mice were exposed to the CB aerosol at 29.33 ± 9.10 mg/m^3^ for 7 and 14 d. Both IL-6 and IL-8 levels were increased in serum and lung homogenates, but we did not found any change of the TNF-α in either serum or lung homogenates. Though the pro-inflammatory cytokine TNF is considered to be an important mediator in inflammation, Saber *et al.* [44] suggested that TNF was not required for the induction of inflammation by CB particles in mice. During the recovery of CB exposure, the IL-8 levels in both serum and lung homogenates were decreased but still higher than the control group, whereas the IL-6 levels were increased in both serum and lung homogenates, compared with the 14 d exposure group. These results suggested that the pro-inflammatory cytokines sustained at a high level for some period induced by CB inhalation even after the cessation of exposure, especially the IL-6 level. Li *et al.* [45] found CB induced neutrophils, lactate dehydrogenase and protein concentration increased in the BALF after 260 nm and 14 nm CB particles were intra-tracheally instilled into rats. The neutrophil influx persisted at least 7 d. The persistent inflammation in lung might contribute to further lung damage, such as fibrosis, hyperplasia, and/or tumor.

The inflammatory response elicited in the lung might be related to the deposition of inhaled PM. We have found that the CB powder was deposited in the lung tissue of mice, including bronchus, bronchiole, bronchioli terminals, and alveoli. The inhalation of particles increased the risk of pulmonary inflammation, which is closely associated with chronic pathological outcomes [8,46,47]. TEM detection indicated that more CB particles were phagocytized by pulmonary macrophage. Some of them were not enveloped with membrane, suggesting that the CB particles could transport through the cell membranes and get into the cells. More deposition of CB particles was found in 14 d exposure group compared with the 7 d exposure group and there were more deposition of CB particles after cessation of exposure for 14 d (Figure 3). The endocytosis (cellular granularity) has been shown to be dose-dependently correlated with pro-inflammatory response induced by CB particles [48], suggesting the importance of the internalized amount of CB in this cellular response.

Immunostaining showed more IL-6 and IL-8 positive cells were from bronchus and alveoli cells. In the recovery group, the bronchus and alveoli cells which deposited CB particles still showed deeply positive staining of IL-6 and IL-8. These phenomena suggested that it is difficult to clean out the CB particles from the lung and might explain the sustained increase of IL-6 and IL-8 levels after CB exposure, even after the cessation of CB exposure. This persistent inflammation in lung induced by CB might contribute to greater genotoxic events. Therefore, the genotoxic effects should be studied in CB-exposed workers in the future study.

## Conclusions

The present study was designed to determine whether lung function deficiency and inflammation were induced by inhalation of CB in an occupationally CB-exposed population and find the relationship of particle retention and inflammation as well as pathology in the lung tissue of mice after inhalation of CB particles. Our data strongly suggest that nanoscale CB particles could damage the pulmonary function and induce pro-inflammatory cytokines secretion in CB-exposed workers. The uptake, deposition, and retention of CB particles in the lung tissue might contribute to inflammation and pathological diseases. These findings provide the first evidence of a link between human exposure to CB and long-term adverse effects on pulmonary function.

## Materials and methods

### Characterization of CB

#### Transmission Electron Microscope (TEM) and Scanning Electron Microscope (SEM)

The size and morphology of CB were measured using a Tecnai G220 TEM instrument (FEI, USA). The nanoparticles dispersed in buffer were cast onto a carbon-coated copper grid sample holder followed by evaporation at room temperature. The surface morphology of CB was observed by a field emission SEM (Sirion 200, FEI, Holland). All samples were sputter-coated with gold before SEM analysis.

#### Brunauer-Emmett-Teller (BET) method

To analyze the pore structure and specific surface characteristics of CB particles, low-temperature (77 K) nitrogen adsorption-desorption isotherms were recorded using an ASAP 2010 from RMIT Applied Chemistry (Micromeritic). The specific surface area was calculated according to the standard BET method in the p/p0 range of 0.05-0.30, where a linear relationship was maintained. N_2_ sorption analysis was performed on a Micromeritics ASAP 2010 porosimeter. Samples were degassed at 60°C under vacuum (p < 10^−2^ Pa) for at least 3 h prior to analysis. Data processing was performed using ASAP 2010 version 5.02 and Origin 7.5 software. Sorption isotherms were measured at −196°C.

### Subjects and sample collection

This study was approved by the Research Ethic Committee of the National Institute for Occupational Health and Poison Control, Chinese Center for Disease Control and Prevention, and informed consent was obtained from each participant. The productive process of CB begins with thermal decomposition of acetylene in the temperature range of 800 to 1800°C. The reaction refers to the equation as follows: C_2_H_2_ = 2C + H_2_. The reactive process doesn’t generate other organic chemicals. All of the 81 male subjects were from the workers packing CB. In this working station, CB was transported by an entirely open system allowing CB particles to be easily dispersed in the workplace. Even though workers wear respiratory protective devices, it is expected that they are a homogeneous group in terms of their dermal and inhalation exposure to this substance. CB-exposed workers had to meet two criteria: (a) male CB packing workers that spend most of their shifts in direct proximity to the CB products, and (b) those working at the spot of the workshop for at least 1 year. A total of 104 healthy unexposed male workers were selected from a water plant and served as the referent group. The recruited controls from the water plant were from the same city as the CB workers, therefore, all the subjects were exposed to the same level of urban air pollution in their spare time. The workers in the control group operate and control pumps at a pump station without processing chlorine disinfection of water. Volunteered CB-exposed workers and age and smoking- matched control subjects were enrolled in September 2012. Exclusion criteria for both CB-exposed and control subjects include history of tuberculosis, pulmonary surgery, cancer, viral myocarditis, congenital heart disease, and recent fever and/or inflammation. All participants signed an informed consent form before commencement of the study. They were then interviewed by trained interviewers using a detailed questionnaire including age, gender, smoking and drinking habits, and working year. Venous blood was collected from each subject after work shift. Based upon company census data, the overall participation rate was 91% for questionnaires and 91% for spirometry.

### Clinical test and pulmonary function tests

The chest radiographs were taken with physical examination after the blood sample collection. Data from the chest radiography and other physical examination were clinically interpreted and reported to the individual worker. Pulmonary function tests (PFTs) were performed before and after the work shift for exposed subjects and once during the post-shift for referent subjects. PFTs were carried out using a portable calibrated vitalograph spirometer (chestac-8800, Japan). Technicians performing the PFTs are certified for completion of the NIOSH cotton dust spirometry course. Testing was performed according to the standards of the American Thoracic Society (ATS). The measured parameters, including FVC, FEV1, FEV1/FVC, MMF, and PEF, were recorded. The pulmonary function values were expressed as percent predicted using the estimated prediction equations as follows: (27.63-0.112 × age) × height for FVC, 34.4 × height-33 × weight −1000 for FEV1, 51 × height/2.54 + 2954–46 × age for MMF and 0.057 × height −0.024 × age +0.225 for PEF. Units are years for age and metres for height. Spirometer calibration was conducted twice a day by the quality assurance team with a 1-liter syringe, in accordance to the spirometer manufacturer’s standard protocol.

### Exposure assessment

To assess the extent to which workers were exposed to CB, standard NIOSH methods were used [20]. Inhalable and respirable dust fractions of CB were measured by personal air samplers carried by 15 volunteers. The atmospheric concentrations of CB were determined in a stationary workplace from 18 samples, and the mean concentration was expressed in mg/m^3^. The sampling time was about 4 h per day. Air samples were collected using BGI 400S personal air sampling pumps (BGI Inc., USA) equipped with a cyclone and a Millipore TEFLON membrane filter (0.1 μm pores). The size of CB in the atmosphere was detected by Aerodynamic Particle Sizer (APS) Spectrometer 3321 (TSI, USA) and the data were analyzed by Aerosol Instrument Manager® software.

### Animals and CB exposure

To study the deposition and pathological changes of lung tissue after CB exposure, the CB aerosol exposure model by dynamic inhalation exposure in mice was established. Briefly, 90 laboratory-bred male BALB/c mice, 9 weeks old, weighed 20 ± 2 g, were procured from the Experimental Animal Center of Vital River Laboratories (Beijing, China). An ambient condition with temperature of 25 ± 2°C, relative humidity of 50 ± 2%, and photoperiod of 12 h was maintained throughout the study. The animals were habituated in the experimental room for 2–3 days. All animals were given access to food and water ad libitum in stainless cages, and they received humane treatment in compliance with the Principles of Laboratory Animal Care formulated by the National Society for Medical Research and the Guide for the Care and Use of Laboratory Animals prepared by the National Academy of Sciences and published by the National Institutes of Health (NIH Publication No. 80–23, revised 1978). The ethical regulations have been followed in accordance with national and institutional guidelines for the protection of animal welfare during experiments.

The mice were randomly divided into four groups, the control group, 7 d exposure group, 14 d exposure group, and the recovery group. The numbers of four groups of animals were 45, 15, 15, and 15, respectively. CB (≥99.8%) was from the CB workplace and its characteristics were the same as the CB to which the workers were exposed. Mice in 7 and 14 d exposure groups were exposed to CB in the inhalation chamber at 30 mg/m^3^ for 6 h/day for a continuous exposure of 7 and 14 d. Mice in the recovery group were exposed to CB for 14 d and recovered for another 14 d. At the same time, the control animals were exposed to filtered air for 6 h/day. The mice in 7 and 14 d exposure groups were sacrificed 24 h after the last exposure. The mice in the recovery group were sacrificed 14 d after the last exposure. Each of the 15 mice in the control group were randomly selected and sacrificed at the same time as the three exposure groups.

All animal exposures were carried out in compartmentalized and horizontal flow, whole-body inhalation chambers (the volume is 45 L). Each chamber can hold up to 45 mice. Total flow through the chambers was ~100 L/min. After exposure, animals were transferred to plastic filter-top cages. The particle-containing atmospheres were generated using a dust generator with a screw feeder (Nine electronics Co., LTD, Guangzhou, China). Aerosolized particle charges were brought to Boltzmann equilibrium by passage through an 85Kr source. Aerosol concentration was continuously monitored by a BGI 400S personal air sampler (BGI Inc., USA) with 7510H electronic flowmeter revising the quantity of flow (Bios, USA). Mass concentration and particle size were periodically measured by gravimetric filter and impactor sampling.

Estimated lung deposition of CB nanoparticles was calculated using the following equation [49]:

$$ \begin{array}{l}\mathrm{Lung}\ \mathrm{burden}=\left(\mathrm{M}\mathrm{V}\right)\times \left(\mathrm{T}\right)\times \left(\mathrm{CON}\right)\times \left(\mathrm{D}\mathrm{F}\right)\\ {}=\left(24\mathrm{mL}/ \min \right)\left(360 \min \right)\left(30\mathrm{mg}/{\mathrm{m}}^3\right)\left(1{\mathrm{m}}^3/1,000,000\mathrm{mL}\right)(0.338)\end{array} $$

where (MV) is the minute ventilation of the exposed animal (mL/min); (T) is the total exposure time (min); (CON) is the average exposure concentration (mg/m^3^); and (DF) is the pulmonary deposition fraction for the alveolar region of the particles inhaled. A minute ventilation of 24 mL/min was used because this value is typical for the average age, weight, and strain of lab mouse used in this study. The value for the DF was extrapolated from validated lung deposition curves for the mouse model [18]. Although there is a degree of uncertainty in the published deposition curves, these data provide the basis for an estimation of the total lung deposition. Therefore, lung deposition of CB nanoparticles was estimated to be 613.2 and 1226.4 μg/mouse after CB exposure for 7d and 14d, respectively.

At the end of the exposure, the animals were anesthetized and then blood samples were collected. Serum was harvested by centrifugation at 700 × g for 10 min. The trachea, lungs, liver, kidneys, and spleen tissues were removed, weighed, and stored in liquid nitrogen for further analysis.

### Hematoxylin-Eosin **(**HE) staining and immunohistochemical staining (IHC)

Six animals in each group were deeply anesthetized with chloral hydrate and perfused with 20 mL of 37°C saline solution from the left ventricle. Mice were then perfused with 100 mL of 4°C 4% paraformaldehyde in a phosphate buffer solution (PBS). The lungs were separated and maintained in a fixative solution (4% paraformaldehyde) until they were embedded with paraffin. Paraffin sections (5 μm thickness) were prepared with routine methods. For pathological changes of lung tissue, the slice was stained with HE using standard procedures. The abnormalities in the lung were determined as described previously, with some modifications [50]. The scoring criteria were as follows: a) alveolar congestion, b) fibrin exudation, c) desquamation of alveolar epithelial cells, d) infiltration or aggregation of neutrophils in airspace or vessel wall, and e) thickness of alveolar wall. Each item was scored on a 5-point scale as follows: 0 = minimal damage, 1 = mild damage, 2 = moderate damage, 3 = severe damage, and 4 = maximal damage.

For IHC, paraffin sections were dewaxed in xylene, rehydrated with distilled water, and then subjected to antigen retrieval for 30 min at 95°C. The slides were subsequently incubated overnight at 4°C with the following antibodies: IL-8 (1:100; Bioworld, Nanjing, China) and IL-6 (1:100, Bioworld, Nanjing, China). Slides were then treated with an anti-rabbit secondary antibody (ZSGB-Biology, Beijing, China) and developed using avidin-conjugated HRP with diaminobenzidine (DAB) as a substrate (ZSGB-Biology), followed by hematoxylin counterstaining. Images were observed under a light microscope (Olympus, Japan).

### TEM image of the lung cell after CB particles exposure

For TEM image, lung tissue was obtained immediately after the animals were sacrificed. Briefly, three animals in each group were deeply anesthetized with chloral hydrate and perfused with 20 mL of 37°C saline solution. Mice were then perfused with 100 mL of 4°C 0.25% glutaraldehyde in 4% paraformaldehyde. The lung was cut into small pieces (1 mm^3^) and fixed by immersion in 2% glutaraldehyde at 4°C, and rinsed in 0.1 M phosphate buffer (pH 7.4), followed by post fixation in 1% osmium tetroxide, block staining in 1% aqueous uranyl acetate, and dehydration using alcohol. The tissues were embedded in 100 EPON for 48 h at 70°C. Ultra-thin sections of 70 nm were cut and stained with 2% uranil acetate and lead citrate, and examined under H-7500 TEM (Hitachi, Japan).

### Cytokines analysis by Cytometric Bead Array (CBA) and enzyme-linked immunosorbent assay **(**ELISA)

For human samples, venous blood was collected and the serum was separated by centrifugation at 700 × g and stored at −80°C until analyzed. The cytokines in human serum were analyzed by CBA. Briefly, IL-1β, IL-6, IL-8, MIP-1β, TNF-α, and MCP-1 concentrations of CB workers and control workers were measured. Human Soluble Protein Master Buffer Kit, Human IL-1β Flex Set (Bead B4), Human IL-6 Flex Set (Bead A7), Human IL-8 Flex Set (Bead A9), Human MCP-1 Flex Set (Bead D8), Human MIP-1β Flex Set (Bead E4), and Human TNF Flex Set (Bead C4) were used according to the manufacturer’s protocol (BD Biosciences). Data were acquired on BD Canto and BD LSRFortessa flow cytometers and analyzed by FCAP Array Software (Soft Flow Inc., Pecs, Hungary).

For animal samples, the blood was collected from angular vein after deep anesthetization with chloral hydrate. The angular vein is a small vein near the eye. The angular vein was pierced by a needle and the blood was collected. Blood and lung homogenate were centrifuged at 700 × g and then the supernatants were collected and stored at −80°C until analyzed. IL-6, IL-8, IL-1β, MIP-1β, and TNF-α were evaluated with commercially available mouse ELISA kit according to manufacturers’ recommendations (IL-6, IL-1β, MIP-1β, and TNF-α kits were from R&D Systems, Minneapolis MN and IL-8 kit was from Cusabio Biotech Company, Wuhan, China).

### Statistical analysis

Natural logarithmic (ln) transformation was applied to cytokines in serum of human samples to satisfy the normal distribution. The quantitative CBA and ELISA data were expressed as mean ± SD. Unless specified, data are representative of the indicated number of subjects or independent experiments. Student’s *t* test or Chi square test were performed to analyze the differences between groups as appropriate. Values of *P <* 0.05 were considered significantly different.

## Additional files

Additional file 1: Table S1. Multiple regression analysis of age, ln(height), standardized weight, smoking status, alcohol use and black carbon exposure on different lung function variables^a^. Additional file 2: Table S2. Lung function parameters stratified by pack-years smoked in the control and CB-exposed groups. Additional file 3: Table S3. Cytokines levels stratified by pack-years smoked in the control and CB-exposed groups (pg/mL). Additional file 4: Figure S1. The body weight of mice after CB exposure for different time and recovery for 14 d after 14 d CB exposure. Data were expressed as mean ± SD. Multi-group comparisons of the means were carried out by a one-way analysis of variance test followed by SNK’s multiple comparison tests. **P <* 0.05 compared to the control group. Additional file 5: Table S4. The organ coefficients after CB exposure in mice (mean ± SD, g/g body weight). Additional file 6: Figure S2. Score of the lung abnormalities of mice after CB exposure for different time and recovery for 14 d after 14 d CB exposure. Data were expressed as mean ± SD. Multi-group comparisons of the means were carried out by a one-way analysis of variance test followed by SNK’s multiple comparison tests. **P <* 0.05 compared to the control group.

## Abbreviations

BET: Brunauer-Emmett-Teller
BMI: Body mass index
CB: Carbon black
CBA: Cytometric bead array
FEV1%: Percent predicted forced expiratory volume in 1 second
FVC%: Percent predicted forced vital capacity
HE: Hematoxylin-eosin
IHC: Immunohistochemical staining
IL-1β: Interleukin 1 β
IL-6: Interleukin 6
IL-8: Interleukin 8
MCP-1: Monocyte chemoattractant protein-1
MIP: Macrophage inflammatory protein
MMF%: Percent predicted maximal midexpiratory flow curve
PEF%: Percent predicted peak expiratory flow
PM: Particulate matter
SEM: Scanning electron microscopy
TEM: Transmission electron microscopy
TLV: Threshold limit value
TNF-α: Tumor necrosis factor α
