# Supplementary material for: Reduced pulmonary function and increased pro-inflammatory cytokines in nanoscale carbon black-exposed workers
Source: Part Fibre Toxicol. 2014 Dec 14;11:73. doi: 10.1186/s12989-014-0073-1 (PMC4318129; doi:10.1186/s12989-014-0073-1)
Supplement: Additional file 1: Table S1. — Multiple regression analysis of age, ln(height), standardized weight, smoking status, alcohol use and black carbon exposure on different lung function variablesa. [file 12989_2014_73_MOESM1_ESM.docx]

| Additional file 1: Table S1. Multiple regression analysis of age, ln(height), standardized weight, smoking status, alcohol use and black carbon exposure on different lung function variables^a^ | | | | | | | | |  |  |
| --- | --- | --- | --- | --- | --- | --- | --- | --- | --- | --- |
| Variables | FVC% | | FEV1% | | FEV1/FVC | | PEF% | | MMF% | |
|  | β (95% CI) | *P*-value | β (95% CI) | *P*-value | β (95% CI) | *P*-value | β (95% CI) | *P*-value | β (95% CI) | *P*-value |
| Age (years) | 0.001 (-0.002 - 0.005) | 0.448 | 0.005 (0.001 – 0.009) | 0.010 | -0.001 (-0.003 - 0.000) | 0.079 | -0.003 (-0.009 - 0.003) | 0.326 | 0.006 (-0.001 - 0.013) | 0.085 |
| Ln(height) (cm) | 2.212 (1.620 – 2.805) | <0.001 | 1.307 (0.679 – 1.935) | <0.001 | -0.120 (-0.335 - 0.095) | 0.271 | 0.420 (-0.515 – 1.356) | 0.376 | 1.593 (0.467 – 2.718) | 0.006 |
| Standardized weight (kg) | -0.023 (-0.045 - -0.001) | 0.041 | -0.018 (-0.041 - 0.005) | 0.132 | 0.004 (-0.004 - 0.012) | 0.299 | 0.011 (-0.023 - 0.045) | 0.529 | 0.011 (-0.030 - 0.052) | 0.598 |
| Smoking status | -0.010 (-0.052 - 0.033) | 0.659 | -0.011 (-0.056 - 0.034) | 0.634 | -0.001 (-0.016 - 0.015) | 0.904 | -0.030 (-0.098 - 0.037) | 0.372 | -0.023 (-0.104 - 0.058) | 0.570 |
| Alcohol use | -0.018 (-0.076 - 0.040) | 0.546 | -0.029 (-0.090 - 0.032) | 0.352 | -0.009 (-0.030 - 0.012) | 0.405 | -0.027 (-0.118 - 0.064) | 0.561 | -0.065 (-0.174 - 0.045) | 0.247 |
| Black carbon exposure | -0.017 (-0.057 - 0.024) | 0.425 | -0.043 (-0.086 - 0.000) | 0.051 | -0.023 (-0.037 - -0.008) | 0.003 | -0.165 (-0.229 - -0.100) | <0.001 | -0.088 (-0.166 - -0.010) | 0.026 |
| ^a^Ln-transformed. |  |  |  |  |  |  |  |  |  |  |
