# Supplementary material for: Reduced pulmonary function and increased pro-inflammatory cytokines in nanoscale carbon black-exposed workers
Source: Part Fibre Toxicol. 2014 Dec 14;11:73. doi: 10.1186/s12989-014-0073-1 (PMC4318129; doi:10.1186/s12989-014-0073-1)
Supplement: Additional file 2: Table S2. — Lung function parameters stratified by pack-years smoked in the control and CB-exposed groups. [file 12989_2014_73_MOESM2_ESM.docx]

| Additional file 2: Table S2 Lung function parameters by pack-years smoked in the control and CB-exposed groups | | | | | | | |
| --- | --- | --- | --- | --- | --- | --- | --- |
| Lung function parameters | Control group | |  | CB-exposed group | | *P*-value^b^ | *P*_interaction_^c^ |
|  | n | mean±SD |  | n | mean±SD |  |  |
| FVC% |  |  |  |  |  |  |  |
| Pack-years=0 | 31 | 104.40±11.40 |  | 22 | 101.32±15.92 | 0.328 | 0.323 |
| 0< Pack-years≤12.5^a^ | 32 | 102.41±19.55 |  | 35 | 101.19±10.53 | 0.875 |  |
| Pack-years>12.5^a^ | 41 | 104.95±12.72 |  | 24 | 98.93±15.06 | 0.085 |  |
| FEV1% |  |  |  |  |  |  |  |
| Pack-years=0 | 31 | 100.70±13.26 |  | 22 | 97.01±14.90 | 0.067 | 0.710 |
| 0< Pack-years≤12.5^a^ | 32 | 99.80±15.46 |  | 35 | 98.30±11.48 | 0.721 |  |
| Pack-years>12.5^a^ | 41 | 101.96±13.87 |  | 24 | 99.22±15.97 | 0.191 |  |
| FEV1/FVC |  |  |  |  |  |  |  |
| Pack-years=0 | 31 | 0.87±0.04 |  | 22 | 0.84±0.05 | 0.016 | 0.127 |
| 0< Pack-years≤12.5^a^ | 32 | 0.88±0.05 |  | 35 | 0.84±0.05 | 0.004 |  |
| Pack-years>12.5^a^ | 41 | 0.86±0.06 |  | 24 | 0.86±0.04 | 0.792 |  |
| PEF% |  |  |  |  |  |  |  |
| Pack-years=0 | 31 | 93.56±17.97 |  | 22 | 81.82±19.93 | 0.032 | 0.701 |
| 0< Pack-years≤12.5^a^ | 32 | 93.39±18.33 |  | 35 | 76.25±14.61 | <0.001 |  |
| Pack-years>12.5^a^ | 41 | 93.03±17.99 |  | 24 | 77.19±16.83 | 0.002 |  |
| MMF% |  |  |  |  |  |  |  |
| Pack-years=0 | 31 | 95.99±20.97 |  | 22 | 86.46±25.43 | 0.094 | 0.848 |
| 0< Pack-years≤12.5^a^ | 32 | 97.05±26.41 |  | 35 | 84.64±22.08 | 0.107 |  |
| Pack-years>12.5^a^ | 41 | 95.70±23.89 |  | 24 | 89.45±24.02 | 0.312 |  |
| ^a^Median of smokers; ^b^Two-sample t test; ^c^Adjustment for age, BMI, and alcohol use. FVC%: percent predicted forced vital capacity, FEV1%: percent predicted forced expiratory volume in 1 second, MMF%: percent predicted maximal midexpiratory flow curve, PEF%: percent predicted peak expiratory flow. | | | | | | | |
