# Supplementary material for: Reduced pulmonary function and increased pro-inflammatory cytokines in nanoscale carbon black-exposed workers
Source: Part Fibre Toxicol. 2014 Dec 14;11:73. doi: 10.1186/s12989-014-0073-1 (PMC4318129; doi:10.1186/s12989-014-0073-1)
Supplement: Additional file 3: Table S3. — Cytokines levels stratified by pack-years smoked in the control and CB-exposed groups (pg/mL). [file 12989_2014_73_MOESM3_ESM.docx]

| Additional file 3: Table S3 Cytokines levels by pack-years smoked in the control and CB-exposed groups (pg/mL) | | | | | | |  | |  |
| --- | --- | --- | --- | --- | --- | --- | --- | --- | --- |
| Cytokines | Control group | |  | CB-exposed group | | *P*-value^b^ | | *P*_interaction_^c^ | |
|  | n | (median, 5%-95%) |  | n | (median, 5%-95%) |  |  |  |  |
| IL-1β |  |  |  |  |  |  | |  | |
| Pack-years=0 | 31 | 3.60 (0.00-119.64) |  | 22 | 13.36 (2.49-56.91) | 0.014 | | 0.490 | |
| 0< Pack-years≤12.5^a^ | 32 | 3.78 (0.00-14.20) |  | 35 | 11.88 (1.88-35.47) | <0.001 | |  |  |
| Pack-years>12.5^a^ | 41 | 5.88 (0.00-26.59) |  | 24 | 12.71 (0.28-49.06) | 0.02 | |  |  |
| IL-6 |  |  |  |  |  |  | |  | |
| Pack-years=0 | 31 | 22.01 (1.85-202.32) |  | 22 | 201.14 (26.52-825.45) | <0.001 | | 0.521 | |
| 0< Pack-years≤12.5^a^ | 32 | 23.70 (2.72-150.81) |  | 35 | 181.51 (52.67-660.75) | <0.001 | |  |  |
| Pack-years>12.5^a^ | 41 | 37.40 (1.37-202.25) |  | 24 | 197.11 (50.80-578.75) | <0.001 | |  |  |
| IL-8 |  |  |  |  |  |  | |  | |
| Pack-years=0 | 31 | 625.82 (154.65-1739.47) |  | 22 | 1073.04 (305.50-4537.05) | <0.001 | | 0.780 | |
| 0< Pack-years≤12.5^a^ | 32 | 781.69 (145.09-2899.30) |  | 35 | 1131.43 (586.42-3915.64) | <0.001 | |  |  |
| Pack-years>12.5^a^ | 41 | 934.76 (149.24-2771.77) |  | 24 | 1517.61 (85.32-3653.10) | 0.001 | |  |  |
| MIP-1β |  |  |  |  |  |  | |  | |
| Pack-years=0 | 31 | 859.92 (287.22-3387.57) |  | 22 | 3223.28 (1462.17-7236.57) | <0.001 | | 0.973 | |
| 0< Pack-years≤12.5^a^ | 32 | 829.38 (207.87-3077.63) |  | 35 | 2352.97 (1205.97-10337.71) | <0.001 | |  |  |
| Pack-years>12.5^a^ | 41 | 755.49 (145.06-2831.65) |  | 24 | 2517.91 (621.00-22743.24) | <0.001 | |  |  |
| TNF-α |  |  |  |  |  |  | |  | |
| Pack-years=0 | 31 | 36.47 (0.00-185.03) |  | 22 | 238.31 (20.81-685.45) | <0.001 | | 0.732 | |
| 0< Pack-years≤12.5^a^ | 32 | 90.91 (0.22-210.33) |  | 35 | 232.13 (76.25-490.89) | <0.001 | |  |  |
| Pack-years>12.5^a^ | 41 | 40.92 (0.00-163.46) |  | 24 | 248.45 (85.53-674.52) | <0.001 | |  |  |
| MCP-1 |  |  |  |  |  |  | |  | |
| Pack-years=0 | 31 | 275.76 (86.89-474.74) |  | 22 | 220.70 (103.74-494.83) | 0.342 | | 0.779 | |
| 0< Pack-years≤12.5^a^ | 32 | 222.35 (57.68-408.78) |  | 35 | 237.33 (66.78-327.69) | 0.967 | |  |  |
| Pack-years>12.5^a^ | 41 | 273.73 (153.53-613.28) |  | 24 | 281.03 (76.54-538.76) | 0.619 | |  |  |
| ^a^Median of smokers; ^b^Two-sample t test; ^c^Adjustment for age, BMI, and alcohol use. | | | | | |  | |  | |
