# Supplementary material for: Reduced pulmonary function and increased pro-inflammatory cytokines in nanoscale carbon black-exposed workers
Source: Part Fibre Toxicol. 2014 Dec 14;11:73. doi: 10.1186/s12989-014-0073-1 (PMC4318129; doi:10.1186/s12989-014-0073-1)
Supplement: Additional file 5: Table S4. — The organ coefficients after CB exposure in mice (mean ± SD, g/g body weight). [file 12989_2014_73_MOESM5_ESM.docx]

Additional file 5: Table S4 The organ coefficients after CB exposure in mice (mean±SD, g/g body weight)

| Tissues | 7d | |  | 14d | |  | Recovery | |
| --- | --- | --- | --- | --- | --- | --- | --- | --- |
|  | Control | CB exposure |  | Control | CB exposure |  | Control | CB exposure |
| Trachea | 0.0017±0.0003 | 0.0021±0.0006 |  | 0.0016±0.0002 | 0.0014±0.0003 |  | 0.0017±0.0005 | 0.0017±0.0005 |
| Lung | 0.0068±0.0005 | 0.0071±0.0009 |  | 0.0062±0.0004 | 0.0077±0.0009^*^ |  | 0.0066±0.0008 | 0.0081±0.0007* |
| Liver | 0.0536±0.0030 | 0.0600±0.0035 |  | 0.0604±0.0088 | 0.0598±0.0024 |  | 0.0566±0.0029 | 0.0562±0.0015 |
| Kidney | 0.0151±0.0006 | 0.0155±0.0010 |  | 0.0147±0.0006 | 0.0153±0.0010 |  | 0.0166±0.0028 | 0.0154±0.0006 |
| Spleen | 0.0042±0.0007 | 0.0035±0.0005 |  | 0.0040±0.0004 | 0.0052±0.0006^*^ |  | 0.0040±0.0007 | 0.0035±0.0006 |

* *P*<0.05 compared with the control.
