# Supplementary material for: Reduced pulmonary function and increased pro-inflammatory cytokines in nanoscale carbon black-exposed workers
Source: Part Fibre Toxicol. 2014 Dec 14;11:73. doi: 10.1186/s12989-014-0073-1 (PMC4318129; doi:10.1186/s12989-014-0073-1)
Supplement: Supplementary file 6 [file 12989_2014_73_MOESM6_ESM.docx]

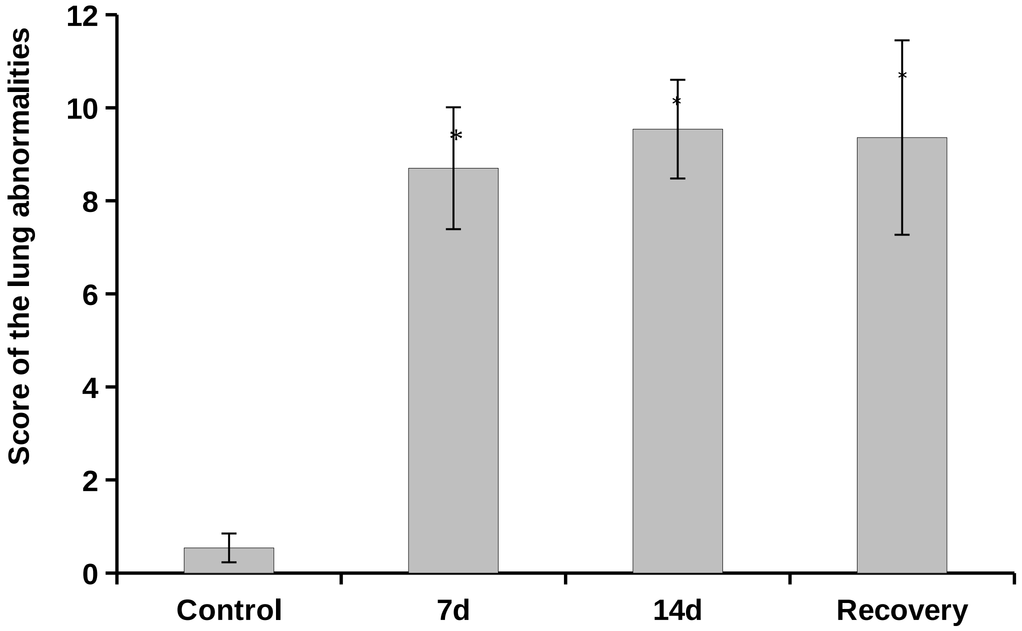

Score of the lung abnormalities
12
10
8
6
4
2
0
*
*
*
Control
7d
14d
Recovery
